# Fake facts and alternative truths in medical research

**DOI:** 10.1186/s12910-018-0243-z

**Published:** 2018-01-27

**Authors:** Bjørn Hofmann

**Affiliations:** 1 The Institute for the Health Sciences, Norwegian University of Science and Technology (NTNU), PO Box 1, N-2802 Gjøvik, Norway; 2 Centre for Medical Ethics, University of Oslo, Oslo, Norway

**Keywords:** Conflict of interest, Polarized research, Mammography screening, Breast cancer, Overdiagnosis, Mortality

## Abstract

**Background:**

Fake news and alternative facts have become commonplace in these so-called “post-factual times.” What about medical research - are scientific facts fake as well? Many recent disclosures have fueled the claim that scientific facts are suspect and that science is in crisis. Scientists appear to engage in *facting interests* instead of *revealing interesting facts*. This can be observed in terms of what has been called *polarised research*, where some researchers continuously publish positive results while others publish negative results on the same issue – even when based on the same data. In order to identify and address this challenge, the objective of this study is to investigate how polarised research produce “polarised facts.” Mammography screening for breast cancer is applied as an example.

**Main body:**

The main benefit with mammography screening is the reduced breast cancer mortality, while the main harm is overdiagnosis and subsequent overtreatment. Accordingly, the Overdiagnosis to Mortality Reduction Ratio (OMRR) is an estimate of the risk-benefit-ratio for mammography screening. As there are intense interests involved as well as strong opinions in debates on mammography screening, one could expect polarisation in published results on OMRR. A literature search identifies 8 studies publishing results for OMRR and reveals that OMRR varies 25-fold, from 0.4 to 10. Two experts in polarised research were asked to rank the attitudes of the corresponding authors to mammography screening of the identified publications. The results show a strong correlation between the OMRR and the authors’ attitudes to screening (*R* = 0.9).

**Conclusion:**

Mammography screening for breast cancer appears as an exemplary field of strongly polarised research. This is but one example of how scientists’ strong professional interests can polarise research. Instead of revealing interesting facts researchers may come to *fact interests*. In order to avoid this and sustain trust in science, researchers should disclose professional and not only financial interests when submitting and publishing research.

**Electronic supplementary material:**

The online version of this article (10.1186/s12910-018-0243-z) contains supplementary material, which is available to authorized users.

## Background

“*Science is built of facts the way a house is built of bricks: but an accumulation of facts is no more science than a pile of bricks is a house*” (Henri Poincaré).

Fake news and alternative facts have become commonplace in these so-called “post-factual times.” What about medical research? Are scientific facts fake as well? A wide range of scientific results have been shown to be false [1]. Even much cited studies don’t hold up and are hard to replicate [2–7]. Initially strong effects of clinical interventions reported in highly cited articles are frequently contradicted [8]. Scientific results are fashioned by who finances research [9] and by researchers’ ties to industry [10]. Spoof research is frequently accepted [11], and scientific truth and objectivity is challenged [12, 13]. All this fuels the claim that scientific facts are suspect and that science is in crisis [14].

One source of crisis in science is when facts are based on confirmative empirical testing [15] or that research hypotheses, models, and approaches are directed by strong interests. The latter can be observed in polarised fields of research. Polarisation occurs when “reputable scientists hold radically opposed views leading to the segregation of the scientific community into groups in part constituted by their opposition to other groups in the field. Polarisation goes beyond mere disagreement. It occurs when researchers begin (1) to self-identify as proponents of a particular position that needs to be strongly defended beyond what is supported by the data and (2) to discount arguments and data that would normally be taken as important in a scientific debate” [16]. In polarised research scientists come to engage in *facting interests* instead of *revealing interesting facts*.

## Main text

How then are we to identify and address such “polarised facts?” One approach is to reveal polarised research fields and to put polarisation on par with other forms of conflicts of interests in scientific publishing. Let me use mammography screening as an example to illustrate how *polarised facts* can be investigated. In this field there are two main points of disagreement: a) What is the benefit of mammography screening, e.g., in terms of reduced breast cancer mortality, and b) what is the harm of this type of screening, e.g., in terms of overdiagnosis? Some researchers tend to claim that the mortality reduction is high, while the overdiagnosis rate is low [17], while others claim that the mortality reduction rate is moderate, while overdiagnosis is high [18]. What is at stake is the risk/benefit-ratio in a utilitarian perspective. Hence, one way to illustrate the polarisation in this field is to scrutinize the divergence in the Overdiagnosis to Mortality Reduction Ratio (OMRR), that is, the ratio of overdiagnosis over the rate of mortality reduction. “Overdiagnosis is the term used when a condition is diagnosed that would otherwise not go on to cause symptoms or death” [19]. Mortality from breast cancer is defined as deaths with breast cancer coded as the underlying cause of death and mortality reduction is defined in terms of reduced breast cancer mortality in a screened group compared to a non-screening group in the assessment of a screening program.

Accordingly, the research questions of this brief study are: What is the OMRR in publicly funded mammography screening programs of women aged 50–69 years old? How is this related to the corresponding authors’ attitudes towards screening? A straight forward literature search identifies 8 studies who have addressed the first question. The studies and their results are shown in Table 1.

**Table 1:** Overdiagnosis to mortality reduction ratio (OMRR) for various studies and the corresponding author’s attitudes to mammography screening as assessed by experts in polarised research (1: Very negative to screening, 2: Negative to screening, 3: Neutral to screening, 4: Positive to screening, 5: Very positive to screening)

Institution/corresponding author	Overdiagnosis to mortality reduction ratio (OMRR)	Attitudes to screening	References
EUROSCREEN group/Dr. Eugenio Paci	4:8 = 0.5	5	[25, 26]
Florentine screening program/Dr. Eugenio Paci	6:10 = 0.6	5	[27]
The Norwegian Research Council (NRC)/Professor Roar Johnsen	5:1 = 5	2	[28]
The Norwegian Breast Cancer Screening Program (NBCSP)/Professor Solveig Hofvind	17:10 = 1.7	5	[29]
Cochrane Collaboration/Director Peter Gøtzsche	10:1 = 10	1	[30, 31]
The Swedish Two-County randomized trial of mammographic screening for breast cancer	4.3: 8.8 = 0.5	4	[17]
The UK Breast Screening Programme in England/Dr. Prue C Allgood	2.3: 5.7 = 0.4		
Marmot report (UK)/Professor Sir Michael Marmot	3:1 = 3	4	[32]
U.S. Preventive Services Task Force (USPSTF)/Dr. Albert L. Siu	19:7 = 2.71	4	[33]

In order to assess the researchers’ attitudes to screening specific questions suggested to identify “polarised conflict of interest.” were adapted to this particular case and were sent to the corresponding authors of the identified publications. However, the corresponding authors found it difficult to answer the questions. As expected, “researchers within a polarised group in a polarised field may not themselves be able to identify the field as polarised or see themselves as belonging to a polarised group”. In order to overcome this problem, two experts on polarised conflict of interest were asked to classify the corresponding authors of the identified publications. Inclusion criteria for these experts were that they were experts on science ethics in general and polarised research in particular, and exclusion criteria were if they had been involved in mammography screening programs or their primary evaluations. The research question and the included articles were not revealed. For a description of the literature search, the questions to the authors, and the questions to the experts, and a discussion of the applied methods, see Additional file 1. The classification of the corresponding interests is given in Table 1.

**Fig. 1:**
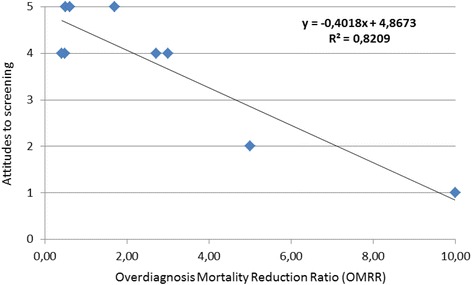
Scatter plot of the relationship between OMRR and attitudes to screening. (1: Very negative to screening, 2: Negative to screening, 3: Neutral to screening, 4: Positive to screening, 5: Very positive to screening)

The correlation between the OMRR and the authors’ attitudes to screening as assessed by experts in polarised research was strong (*R* = 0.9). The scatter plot is shown in Fig. 1.

This indicates that research results in this field are strongly formed by professional interests and attitudes to screening. The same effect of “facted interests” may be observed in other fields of polarised research, and in-depth studies are needed and encouraged.

Of course, personal interest can be a good thing in science. It can motivate important and ground-breaking research. However, it can also bias judgments, cloak influences, direct methodological choices, and skew the presentation of results. Moreover, *framed facts* can influence important health policy decisions. Accordingly, it is crucial to acknowledge that this represents “genuine conflicts of interest” threatening “the objectivity of science” [12] and trust in science. This can be done by a) making researchers state their “polarised conflict of interest” when submitting manuscripts, b) making reviewers explicitly assess polarisation, and c) apply external experts to assess polarisation when reviewers (and/or editors) are too ingrained in the research to be able to make the assessment.

Polarisation may be a general trend resulting from disagreements on research methodology or assessment of evidence (according to GRADE or other). However, it may also result from self-interest [20], intellectual laziness [21, 22], mental shortcuts, or hyper-partisanism [23]. Moreover, emotional conflicts of interests are more difficult to handle than financial conflicts [24].

While philosophers of science and sociologists long have revealed the challenges of value-laden facts and underscored the constitutive value of *disinterestedness* in science [12], it is high time we scientists acknowledge this in practice.

## Conclusion

Scientists appear to engage in *facting interests* as much as in revealing *interesting facts*. Published research on mammography screening for breast cancer illustrates the problem of science being directed by strong professional interests, where some researchers continuously publish positive results while others publish negative results on the same issue – even when based on the same data. Analysing this as polarised research may provide a way to address an important issue threatening to undermine trust in scientific results and medical researchers. Hence editors should a) make researchers state their “polarised conflict of interest” when submitting manuscripts, b) make reviewers explicitly assess polarisation, and c) apply external experts to assess polarisation when reviewers (and/or editors) are too ingrained in the research to be able to make the assessment.

How exactly to assess polarised conflict of interest may need more elaboration and collaborate work. However, Table 2 suggests some questions to ask when assessing polarised conflict of interest. This is a first step illustrating methodological and empirical feasibility.

**Table 2:** Relevant questions to ask when assessing polarised conflict of interest

Addressee	Question
Editors	Is the topic or the field of the submitted manuscript subject to significant controversy (with respect to methods, results, conclusions, or recommendations)?
	Which are the groups (the “poles”) and what do they disagree on?
	Where does the manuscript lie with respect to these groups (poles)?
	Do the suggested or considered reviewers belong to the same pole as the authors?
	Can you find qualified reviewers that are independent of the identified groups?
	Do the authors state their polarised conflict of interest?
	Do you or co-editors have a specific stance on the controversy? If yes, how will you handle this? (stating conflict of interest, using alternative editors etc)
Reviewers and Editors	Based on your expertize in this field, are there groups with competing views on methods, theories, outcomes, or/and policies in the field (of the manuscript)? (Polarisation awareness)
	If yes, do you and the author(s) belong to the same group? (Polarisation idenfitication)
	Based on your reading of the manuscript, if *the results, conclusion or recommendations* of the study were the opposite (data and methods being the same) would you assess the manuscript differently? (Own stance in polarisation)
Researchers	“If the results of your current (well planned and well conducted) project point in the opposite direction of the results of your previous research on this topic, would your first reaction be to reanalyse the data and reconsider your methods, or to reconsider your previous conclusions?” (Result polarisation)
	“If your findings were the exact same as the opposing researchers in this field of research, would your policy recommendations be any different from the recommendations of the opposing group?” (Interpretation polarisation)
	When calculating outcome measures from your results (e.g., risk/benefit ratios) and these result from the methods, models or evidence criteria that you use, would you still use the same methods, models or evidence criteria if the outcome measures were very different (opposing)? (Methods polarisation)
	Is your institution, department, or organization is providing services related to your research? If yes, do you find it appropriate to proclaim “nothing to declare” in the conflict of interest statement? (Affiliation polarisation)

## Additional file

Additional file 1: Detailed informatin on method and discussion of method and results. (PDF 145 kb)

## Abbreviations

EUROSCREEN: The European screening network
NBCSP: The Norwegian breast cancer screening program
NRC: The Norwegian research council
OMRR: Overdiagnosis to mortality reduction ratio
USPSTF: U.S. Preventive Services Task Force
