# Supplementary material for: Fake facts and alternative truths in medical research
Source: BMC Med Ethics. 2018 Jan 27;19:4. doi: 10.1186/s12910-018-0243-z (PMC5787277; doi:10.1186/s12910-018-0243-z)
Supplement: Additional file 1: — Detailed informatin on method and discussion of method and results. (PDF 145 kb) [file 12910_2018_243_MOESM1_ESM.pdf]

Additional file 1:

Fake facts and alternative truths in medical research BMC Medical Ethics

Methods

In order to address the research question a standard literature search in PubMed was performed. Search included all studies until February 1 2017. The search strategy was: (“Overdiagnosis to mortality reduction ratio” OR “benefit-detriment ratio” OR “benefit to detriment ratio” OR “benefit to harm ratio” OR “the ratio between benefits and harms”) AND (mammography OR mammographic OR “breast cancer”) AND screening.

Inclusion criteria were: Studies of publicly funded mammography screening programs, with women of age 50-69 years old.

Exclusion criteria were studies not including overdiagnosis as harm, or reduced mortality rate as benefit.

Identified studies are presented in Table 1 of the article.

In order to identify “polarised conflict of interest” two questions are suggested by Ploug and Holm [16]:

“1. If the results of your current (well planned and well conducted) project point in the opposite direction of the results of your previous research on this topic, would your first reaction be to reanalyse the data and reconsider your methods, or to reconsider your previous conclusions? 2. If your findings were the exact same as the opposing researchers in this field of research, would your policy recommendations be any different from the recommendations of the opposing group?” [16]

In order to make the question more comprehensible in this context, these questions were modified slightly:

1. If the results of the work with this article would have pointed in the opposite direction of the results of your previous research on this topic, would your first reaction be to reanalyse the data and reconsider your methods, or to reconsider your previous conclusions?

2. If your findings in this study were the exact same as the opposing researchers in this field of research, would your policy recommendations be any different from the recommendations of the opposing group?

The questions were sent to the corresponding authors of the identified studies by e-mail, and they were asked to address the questions with respect to the specific study. Four of the eight corresponding authors responded and their answers were very divergent and not conclusive with respect to polarisation. As one of the authors replied: “I consider it difficult to answer yes or no”.

Moreover, some of the included studies were reviews, making the questions less clear. Several of the studies were also the outcome of group work or committee work. Some of the committee members were selected because they had not published on the topic before. Hence, although the suggested questions appeared reasonable to identify polarised conflict of interests, they did not work well in

this case. As also acknowledged by Ploug and Holm themselves: “researchers within a polarised group in a polarised field may not themselves be able to identify the field as polarised or see themselves as belonging to a polarised group.”[16]

In order to overcome this problem, two experts on polarised conflict of interest were selected by approaching scholars who had published on this type of conflict of interest. Inclusion criteria were that they were experts on science ethics in general and polarised research in particular, and exclusion criteria were if they had been involved in mammography screening programs or their primary evaluations. These experts were asked to classify the risk of polarized conflict of interest of the corresponding authors of the identified publications on a 5-level Likert scale. 1: Very negative to screening, 2: Negative to screening, 3: Neutral to screening, 4: Positive to screening, 5: Very positive to screening.

The experts approached each other, deliberated on the ranking, and sent a joint ranking as a response.

Discussion of method

One challenge with the approach of this study lies in the fact that only the corresponding authors were assessed by the experts. They may not be representative for the rest of the authors. One could of course assess all co-authors (or all committee members in external committees) and make a joint score for the whole group. Applying the corresponding author is pragmatic and assumes that the person is a suitable representative of the group, but not that the person has any special influence or power. Addressing the robustness of the methodology is beyond the scope of this *Debate article*, but is a viable and important next step in the study polarised research.

Another challenge is that there is a bias in the selection of experts on polarised conflict of interest. Moreover, one could wish there to be more (than two) included experts. However, given the inclusion and exclusion criteria, the pool of experts in this field is very small. Luckily, both approached experts agreed to participate. Hopefully, the number of experts in this field will be growing, so that more thorough studies can be performed in the future. Accordingly, more sophisticated and robust ranking systems may be applied when more experts on polarised research become available. As such, this study demonstrates a feasible approach to be improved by further research.

Yet another problem is that the experts may assess the risk of polarised conflict of interest on basis of the published outcomes (OMRR) of the researchers. This would make the empirical findings self-reinforcing and the argument circular. However, the experts were asked to assess the corresponding author on basis of the definition of polarised conflict of interest (identification with a specific position and apparent discounting of arguments and data) and not based on specific outcomes. This is in principle independent of OMRR. Nonetheless, one would expect that scholars associated with one pole publish results contrary to those on the other pole (low versus high OMRR).

Moreover, implicitly this study argues that we should be critical to experts in any specific scientific field and investigate their “polarised conflict of interest.” This is of cause also relevant for the author of this study and for the selected experts on polarised research. Is the author “polarised” when analyzing the data or selecting experts? Are the experts really neutral as their ranking may result from biases, preferences, and misconceptions? However important, this is a general problem, as it is

difficult to find any expert that could be declared as fully objective and impartial. One may have “second order polarisations,” i.e., polarised conflict of interest amongst experts on polarised research. The point here is neither that one should ignore nor be paralyzed by such problems. By presenting assessments of polarised conflict of interest they are open for discussions and criticism. Trying to uncover covert phenomena and to make undeclared conflict of interests visible is important. No doubt, the methods for doing so may not be perfect, and may certainly need refinement. However, we should not let the lack of perfection in method stall our focus on an important issue for the application of scientific results as well as trust in science. Hopefully, this initial study will spur fruitful debates and further research on this issue. Hence, it is certainly difficult to find an Archimedean point, but this is the core of the problem in polarized research. If such a point could be found, polarisation would be undermined and the problem resolved.

One may also argue that the questions sent to the authors should not be mentioned in this study, as the responses were not of any value for the study as such. However, the reason for reporting on this is that it illustrates that even though the suggested questions to identify polarised conflict of interest [16] appear reasonable (and can be easily adapted), they may not be very useful in practice. It may make future researchers on polarised research aware of and avoid such practical problems. Besides, reporting on such failures avoids reporting bias (only reporting positive findings).

Another relevant critique of this study is that the definitions of overdiagnosis and mortality reduction vary in the included studies. Those that are in favor of screening define and measure overdiagnosis and mortality reduction differently from those that are less in favor. However, this is exactly at the core of the problem: polarised interests make researchers use different models and methods. Models, outcome measures, time periods, estimates, inclusion- and exclusion criteria, corrections etc are chosen not only based on scientific merits, but also on professional interests.
